# Gallic Acid Mitigates 5-Hydroxymethylfurfural Formation while Enhancing or Preserving Browning and Antioxidant Activity Development in Glucose/Arginine and Sucrose/Arginine Maillard Model Systems

**DOI:** 10.3390/molecules27030848

**Published:** 2022-01-27

**Authors:** Thaísa Abrantes, Nathália Moura-Nunes, Daniel Perrone

**Affiliations:** 1 Laboratório de Bioquímica Nutricional e de Alimentos, Chemistry Institute, Federal University of Rio de Janeiro, Av. Athos da Silveira Ramos 149, CT, Bloco A, Sala 528A, Rio de Janeiro 21941-909, Brazil; abrantesthaisa06@gmail.com; 2 Laboratory of Food Science, Department of Basic and Experimental Nutrition, Nutrition Institute, Rio de Janeiro State University, Rua São Francisco Xavier 524, Pavilhão João Lyra Filho, 12° Andar, Bloco D, Sala 12002, Rio de Janeiro 20559-900, Brazil

**Keywords:** maillard reaction, melanoidins, phenolic compounds, food thermal processes, FRAP, TEAC, HMF

## Abstract

The current trend of lowering 5-hydroxymethylfurfural (5-HMF) dietary exposure is challenging since its formation is parallel with the development of food color, flavor and aroma. We aimed to investigate the effect of gallic acid (GA) addition on 5-HMF formation, color development and antioxidant activity (AA) in a series of Maillard Reaction (MR) model systems. The effects of GA addition on browning and AA development were not uniform for all model systems, but always occurred in the same direction, indicating that these phenomena were interconnected. GA mitigated 5-HMF development in four of the nine tested systems, possibly by preventing the oxidation of MR intermediates. Correlation analysis indicated that when GA addition mitigated 5-HMF formation, browning was either promoted or not affected. The proposed strategy was effective for glucose/arginine and sucrose/arginine systems, since GA mitigated 5-HMF formation (49% and 54%, respectively) in addition to increasing color development and antioxidant activity.

## 1. Introduction

Baking, roasting and frying are examples of thermal processes often used to obtain safe food products with a long shelf life and good sensory quality. In addition to destroying microorganisms, inactivating enzymes, and lowering water activity, these processes also improve color, flavor, aroma and texture of food. At the same time that heating processes may result in the formation of compounds that show positive health effects [1], it causes loss of nutritional value and leads to the formation of harmful compounds with mutagenic, carcinogenic or cytotoxic effects, such as furfurals [2].

The furanic compound 5-hydroxymethylfurfural (5-HMF), an intermediate of both caramelization and Maillard Reaction (MR), has gained much interest from the scientific community due to its high toxicological potential as well as its wide occurrence, being considered to be one of the most important heat-induced contaminants in food, especially coffee, bread, dried fruit, fruit juices, vinegar and honey [3]. Although 5-HMF toxicity in humans is still not irrefutable, its reduction in foods aiming at lowering exposure has become a trend to ensure food safety [4]. Developing food color, flavor and aroma without forming HMF during heating is challenging because both processes share the same precursors and formation pathways [2]. To date, existing strategies to mitigate HMF levels in food can be classified as removal or prevention interventions. Elimination of HMF already formed in the finished product may occur by volatilization (e.g., cooking on open vessel) or decomposition (e. g. ionizing radiation and fermentation). Preventive actions include changes in precursors or pathways to make the reaction conditions less favorable to HMF formation during heating [5]. Modulation of the time/temperature binomial and replacement of reducing sugars by non-reducing ones or polyalcohols have already been tested without success, since HMF formation was not prevented, or food products did not develop color [2].

The preventive strategy of adding compounds that inhibit 5-HMF formation or compete with its precursors has also been investigated [5]. Phenolic compounds, for instance, may play a role in MR together with reducing sugars and amino acids, classically known to be involved in this reaction, altering the formation of Maillard reaction products (MRP). During coffee and bread heat processing, phenolic compounds were found linked to melanoidins, demonstrating their involvement in MR [6,7]. In model systems, the effect of adding isolated phenolic compounds on MRP formation have been studied. Ellagic, gallic, ferulic and syringic acids inhibited the formation of pyrazine and pyridine derivatives [8] and ferulic acid also controlled the formation of early MRP [9], but neither study analyzed 5-HMF. This compound was, in turn, investigated by other authors. Epicatechin quenched 3-deoxy-2-hexosulose and therefore decreased 5-HMF formation [10], whereas chlorogenic acid caused an increase in 5-HMF development [11]. Even though several phenolic compounds mitigated the formation of furan derivatives, including 5-HMF [12], their effect on color development was not investigated. These controversial results may be related to the different complexity and/or composition of the model systems employed.

In this scenario, a comprehensive and systematic approach, using model systems with different combinations of sugars and amino acids, would be relevant to study the effect of adding a phenolic compound on MR modulation and 5-HMF mitigation. To contemplate both reducing and non-reducing sugars, glucose (the most common monosaccharide), fructose (widely distributed in food), and sucrose (the most used disaccharide in food formulation) were chosen. Glycine, lysine and arginine were selected because the former is the simplest amino acid, and the others are highly reactive ones. Finally, considering that the efficiency of polyphenolic compounds in reducing furan derivatives rely on the number of phenolic groups [12], gallic acid (GA) was chosen due to its structure and wide distribution in nature. Therefore, studying GA potential effect on 5-HMF mitigation in such model systems can help to understand how its addition would affect different food matrices. Thus, we aimed at investigating the effect of GA addition on 5-HMF formation, color development and antioxidant activity in a series of MR model systems.

## 2. Results and Discussion

### 2.1. Gallic Acid Participates in the Maillard Reaction Affecting Antioxidant Activity and Browning

**Figure 1:**
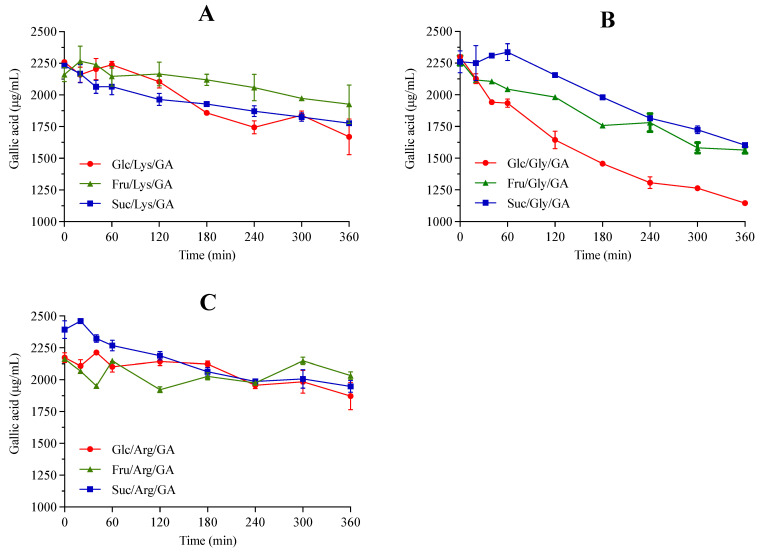
Gallic acid (GA) participated in the Maillard Reaction, being progressively consumed in sugar–amino acid model systems containing glucose (Glc), fructose (Fru) or sucrose (Suc) and lysine (Lys, (**A**)), glycine (Gly, (**B**)) or arginine (Arg, (**C**)).

GA was consumed in all model systems as the reaction progressed (Figure 1), especially in those containing glycine (from 29.1% to 50.2%) and at a lower extent in those containing arginine (from 6.0% to 18.6%). GA concentration did not change in the blank system (Supplementary Figure S1), which was prepared by heating a gallic acid solution under the same conditions used for model systems (heating at 125 °C under reflux for 360 min, pH = 5.0). Considering these results, it is likely that GA participated in the MR rather than being thermally degraded. In fact, in complex systems, such as coffee [13] and bread [7], phenolic compounds participate in MR, being incorporated into melanoidins backbone.

**Figure 2:**
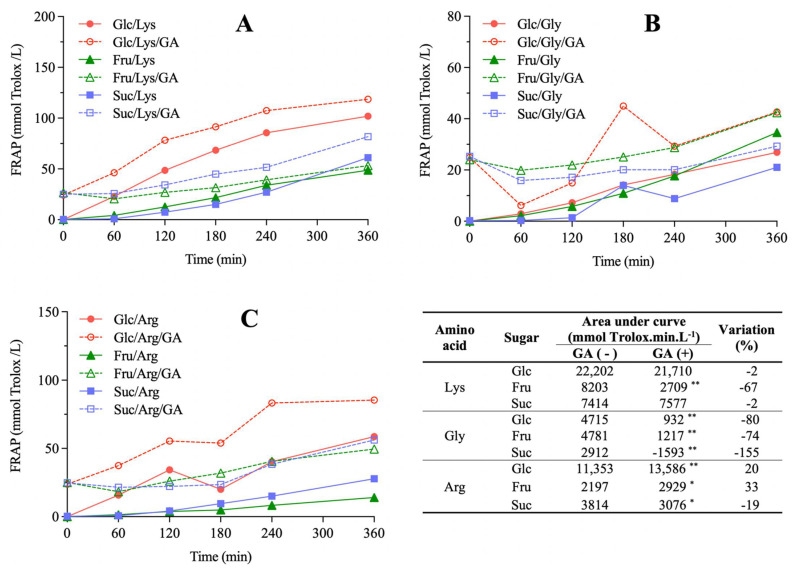
The effect of gallic acid (GA) addition on the antioxidant activity development, measured by the FRAP assay, in Maillard sugar–amino acid model systems containing glucose (Glc), fructose (Fru) or sucrose (Suc) and lysine (Lys, (**A**)), glycine (Gly, (**B**)) or arginine (Arg, (**C**)). Area under curve (AUC) values of AA development and their percent variation due to GA addition are presented in the table. Statistical differences between model systems not added (−) and added (+) with GA were evaluated by Student’s unpaired *t*-test (** *p* < 0.01, * *p* < 0.05).

**Figure 3:**
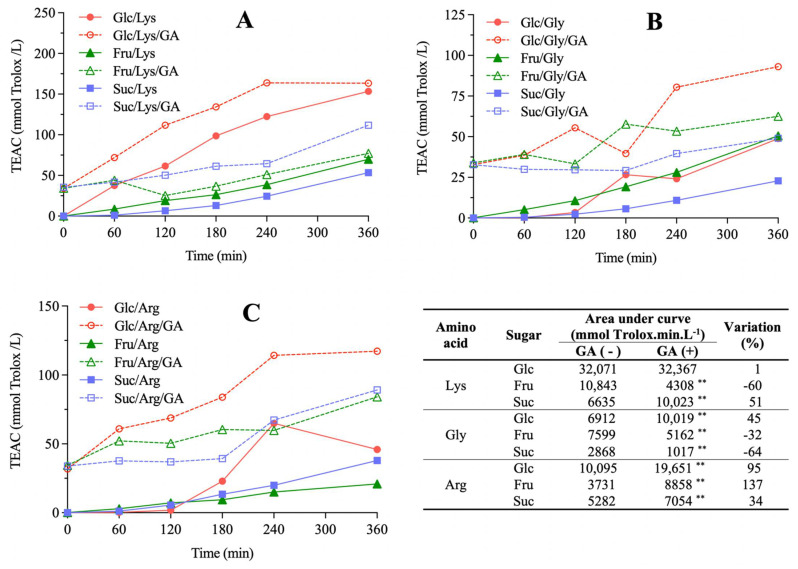
The effect of gallic acid (GA) addition on the antioxidant activity development, measured by the TEAC assay, in Maillard sugar–amino acid model systems containing glucose (Glc), fructose (Fru) or sucrose (Suc) and lysine (Lys, (**A**)), glycine (Gly, (**B**)) or arginine (Arg, (**C**)). Area under curve (AUC) values of AA development and their percent variation due to GA addition are presented in the table. Statistical differences between model systems not added (-) and added (+) with GA were evaluated by Student’s unpaired *t*-test (** *p*<0.01).

AA evaluated by FRAP (Figure 2) and TEAC (Figure 3) assays increased as a function of heating time for all model systems, which is in accordance with previous studies [14,15]. Considering model systems not added with GA, glucose/lysine showed the highest AA values independently of the assay (*p* < 0.05). The antioxidant activity exhibited by GA alone (Supplementary Figure S1), which did not change over time in the blank system, is the reason AUC calculation of FRAP and TEAC was performed for model systems considering the initial values as baseline, thus avoiding overestimated results. Taking this information into account, the effect of GA addition on AA development was distinct among model systems (Tables inserted in Figure 2 and Figure 3). In general, GA addition decreased AA development of model systems containing fructose and glycine (from 32% to 155%), whereas those containing arginine usually showed increased AA development (from 20% to 137%) (*p* < 0.05).

**Figure 4:**
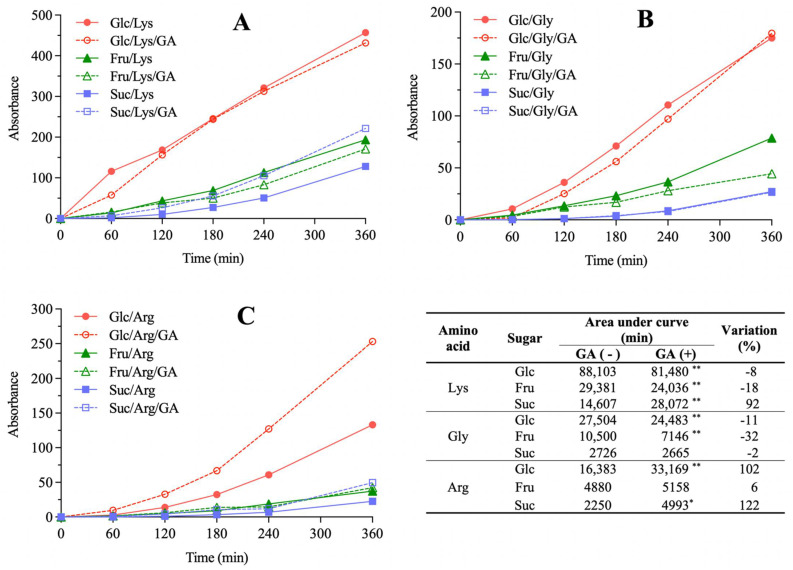
The effect of gallic acid (GA) addition on color development in Maillard sugar–amino acid model systems containing glucose (Glc), fructose (Fru) or sucrose (Suc) and lysine (Lys, (**A**)), glycine (Gly, (**B**)) or arginine (Arg, (**C**)). Area under curve (AUC) values of browning development and their percent variation due to GA addition are presented in the table. Statistical differences between model systems not added (−) and added (+) with GA were evaluated by Student’s unpaired *t*-test (** *p* < 0.01, * *p* < 0.05).

All model systems showed progressive browning as a function of heating time, as expected (Figure 4). Considering model systems not added with GA, the highest color development was observed for model systems with glucose, followed by those prepared with fructose and sucrose, in accordance with the literature [16,17]. Considering amino acids, model systems prepared with lysine showed the highest browning, followed by those prepared with glycine and arginine.

GA addition caused diverse effects on browning (Table inserted in Figure 4). In the glucose/glycine system and in systems containing fructose with glycine or lysine, GA addition decreased color development (from 11% to 32%). Silván et al. [9] also observed reduction in browning (~28%) upon addition of ferulic acid in fructose/protein model systems. On the other hand, GA addition increased color development of the glucose/arginine, sucrose/arginine and sucrose/lysine models (from 92% to 122%) and did not affect glucose/lysine, fructose/arginine and sucrose/glycine models.

Browning positively correlated with AA development measured by FRAP (r = 0.92, *p* < 0.001, *n* = 9) and TEAC (r = 0.97, *p* < 0.001, *n* = 9) assays. It is explained by the formation of melanoidins, the major brown compounds which are referred as the main contributors to AA in MR [18], although mechanisms are still not fully elucidated. The most accepted theories which explain melanoidins AA are based on metal chelation, reactive oxygen species trapping and formation of inactive complexes due to capture of electrophilic species [19]. This relation between browning and AA development has already been established in other model and real food systems [20,21,22].

Upon GA addition, the only model system that exhibited a statistically significant increase in both browning and AA development was glucose/arginine (*p* < 0.0103), whereas fructose/glycine was the only system in which both these parameters decreased (*p* < 0.0008). Nevertheless, positive correlations were observed between the absolute variations in AUC of browning and FRAP (r = 0.68, *p* = 0.044, *n* = 9) and of browning and TEAC (r = 0.72, *p* = 0.028, *n* = 9) due to GA addition, indicating that this phenolic compound caused the same effect in these parameters. In other words, even though the effects of GA addition on browning and AA development were not uniform for all model systems, with some presenting increases and other decreases depending on the sugars and amino acids involved, these effects always occurred in the same direction. Considering that the MR pathways related to the formation of colored and of antioxidant compounds are interconnected, the observed behavior indicates that GA participates in both pathways. Their inhibition in model systems due to GA addition may be explained by its redox reaction with the α-dicarbonyls formed in the intermediate stages of the MR, producing reduced compounds that cannot take part in more advanced stages, in which colored and/or antioxidant MRPs are formed. In fact, GA, catechin and quercetin exhibited over 80% inhibitory effects on the formation of α-dicarbonyl compounds in a glucose/albumin model system [23]. On the other hand, GA exhibit pro-oxidative properties depending on reaction conditions [24]. This may explain the promotion of browning and AA development in model systems upon GA addition. Zhang et al. [11] reported an increase in MR intermediates formation in model systems upon chlorogenic, caffeic and *p*-coumaric acids addition. Similar results were also described for biscuits added with GA, for which an increase in 3-deoxyglucosone was observed [25].

### 2.2. Gallic Acid Addition Mitigates 5-Hydroxymethylfurfural Formation without Compromising Browning in Glucose/Arginine and Sucrose/Arginine Model Systems

**Figure 5:**
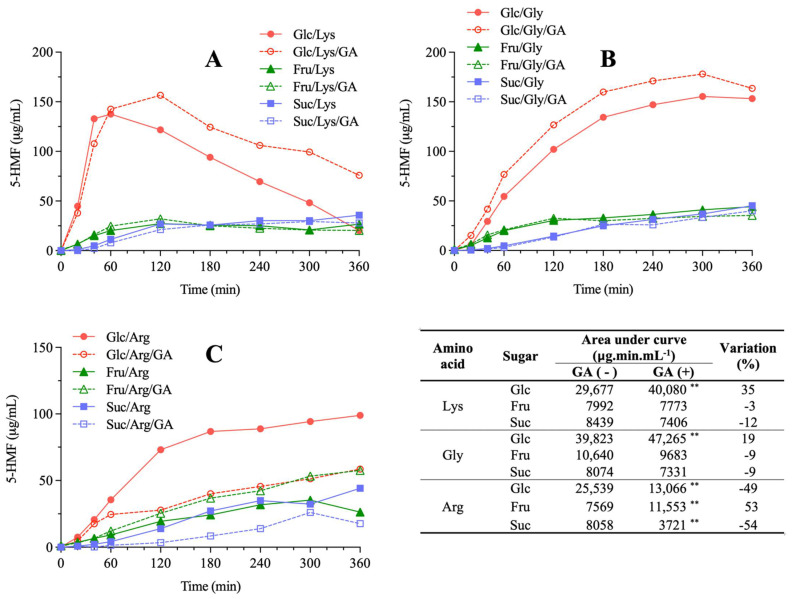
The effect of gallic acid (GA) addition on 5-hydroxymethylfurfural (5-HMF) development in Maillard sugar–amino acid model systems containing glucose (Glc), fructose (Fru) or sucrose (Suc) and lysine (Lys, (**A**)), glycine (Gly, (**B**)) or arginine (Arg, (**C**)). Area under curve (AUC) values of 5-HMF formation and their percent variation due to GA addition are presented in the table. Statistical differences between model systems not added (−) and added (+) with GA were evaluated by Student’s unpaired *t*-test (** *p* < 0.01).

In general, sugars influenced both the amount and rate of 5-HMF formed during the MR in model systems (Figure 5). The highest amounts of this compound were observed in systems containing glucose (their AUC were on average 3.7 times higher than those containing fructose or sucrose). The rate of 5-HMF formation in the first 60 min of reaction was the highest in systems containing glucose (average rate of 1.26 µg/mL/min), followed by those with fructose (average rate of 0.27 µg/mL/min) and sucrose (average rate of 0.11 µg/mL/min). It is known that glucose is a potent promotor of the MR since it is a precursor of dicarbonyl compounds such as 3-deoxyglucosone, which in turn may be transformed into 5-HMF through β-elimination of water under acidic conditions [26]. Amino acids affected the amount of 5-HMF formed only in systems containing glucose, with the highest quantity observed in the glucose/glycine system. Moreover, amino acids affected the rate of 5-HMF development, with faster formation in systems containing lysine (average rate of 0.94 µg/mL/min), followed by glycine (average rate of 0.44 µg/mL/min) and arginine (average rate of 0.27 µg/mL/min) (Figure 5). Lysine favors the development of MR and the formation of its products due to its high reactivity and greater susceptibility to carbonyl-amino reaction [27]. The model system containing glucose and lysine presented a unique behavior in terms of 5-HMF formation with maximum concentrations at around 60 min of reaction, probably explained by the combination of their high reactivities towards MR, and decreasing concentrations thereafter, possibly due to further reactions of HMF with amino acids [28].

GA caused diverse effects on 5-HMF development (Table inserted in Figure 5). Some studies reported that the structures and concentrations of phenolic acids added in the Maillard Reaction caused inhibition and promotion effect. In glucose/lysine, glucose/glycine and fructose/arginine model systems, GA addition increased 5-HMF formation (from 19% to 53%). Depending on concentration, GA may enhance the oxidant activity and promote the formation of MR intermediates [29]. Zhang et al. [11] reported that chlorogenic acid led to a 9% increase in 5-HMF in a model system containing fructose and aspartic acid at pH 5.5, which was attributed to an enhancement of 3-deoxosone formation, a key MR intermediate [30]. In our study, GA mitigated 5-HMF development in all systems containing sucrose and in the glucose/arginine system (up to 54% decrease). The possible mechanism for 5-HMF mitigation involves GA ability to prevent the oxidation of MR intermediates [25]. In fact, it has already been demonstrated that GA is able to inhibit the formation of free radicals from 1,4-pyrazine cation in a model system containing glucose and aminobutanoic acids [8]. Moreover, it is established that in general, phenolic compounds present dicarbonyls-trapping capacity and antioxidant activity through free radical scavenging and metal ion chelation [24]. Recently, Albouchi & Murkovic [12] investigated the mitigation effects of several phenolic compounds on furan derivatives, including 5-HMF. Dry model systems of increasing complexity were studied, including one composed of ground coffee beans. GA mitigated 5-HMF formation by approximately 90% in the least complex dry system, which was composed of sucrose and alanine. Even though this system resembles those investigated in our study in terms of sugar and amino acid composition, one must consider that the higher water activity of our systems decreases the rate of MR.

Absolute variations in 5-HMF levels and color development caused by GA addition were negatively correlated (r = −0.74, *p* = 0.022, *n* = 9), indicating that this phenolic compound caused opposite effects in these parameters. In other words, when GA addition mitigated 5-HMF formation, browning was either promoted or not affected. Glucose/arginine and sucrose/arginine systems stood out in terms of 5-HMF mitigation, 49% and 54%, respectively, in addition to showing increased color development (102% and 122%, respectively) and antioxidant activity, especially when measured by TEAC assay (95% and 34%, respectively).

Extrapolating our results to food products, one could hypothesize that GA addition could mitigate 5-HMF formation in arginine-rich matrixes, such as nuts and seeds, meat products, legumes and potatoes [31,32,33,34,35]. Prior to heat processing of these foods, GA could be added to their surface, either by immersion on a GA solution or by rubbing a powder/spice mix containing this phenolic acid. It should be noted, however, that there is evidence in the literature indicating that mitigation effectiveness decreases with increasing system complexity. For instance, GA had no effect on 5-HMF mitigation in a biscuit model system [25] and its mitigation efficiency was completely lost in a coffee system compared to model systems [12].

## 3. Materials and Methods

### 3.1. Solvents, Reagents and Standards

Acetonitrile, L-arginine hydrochloride, formic acid, glacial acetic acid and methanol were purchased from Tedia (Fairfield, OH, USA). Anhydrous sodium acetate, 2,2-azino-bis(3-ethylbenzthiazoline-6-sulfonate (ABTS), D-fructose, gallic acid monohydrate, L-glycine, D-glucose, 5-hydroxymethylfurfural, 6-hydroxy-2,5,7,8-tetramethylchroman-2-carboxylic acid (Trolox), potassium persulfate, D-sucrose and 2,4,6-tripyridyl-1,3,5-triazine (TPTZ) were purchased from Sigma-Aldrich Chemical Co. (St. Louis, MO, USA). L-lysine was purchased from Bioworld Fine Chemical (Dublin, OH, USA). Hydrochloric acid (fuming 37%) was purchased from Merck KGaA (Darmstadt, Germany). Iron chloride hexahydrate was purchased from Vetec Química Fina Ltd.a. (São Paulo, Brazil). Ultrapure Milli-Q water (Millipore, Bedford, MA, USA) was used throughout the experiments. All chemicals were of analytical grade.

### 3.2. Preparation of Maillard Model Systems

Model systems were prepared according to Bailey et al. [36] with modifications. Stock solutions of glucose, fructose, sucrose, glycine, lysine and arginine (1.25 mol/L), as well as of gallic acid (0.05 mol/L) were prepared in sodium acetate buffer (1.0 mol/L) and their pH was adjusted to 5.0 with glacial acetic acid. In a flat-bottomed flask, sugar and amino acid solutions (10 mL of each) were mixed with gallic acid or acetate buffer (5 mL). In this way, 18 different Maillard model systems, nine with and nine without GA were prepared, with final concentrations of 0.5 mol/L of sugars and amino acids and 0.01 mol/L of gallic acid, when present. Then, solutions were heated under reflux in a mineral oil bath (125 °C) with magnetic agitation for 360 min. A blank system, containing only GA in acetate buffer was prepared and heated at the same conditions. All sample aliquots collected were cooled into an ice bath before analysis.

Browning reproducibility was assessed in triplicates of glucose/lysine and sucrose/glycine model systems, which showed the highest and the lowest browning intensity, respectively, in previous tests. Both model systems showed good reproducibility with coefficient of variation of up to 5%. Since browning represents MR development as a whole, this parameter was considered an appropriated surrogate to evaluate the reproducibility of 5-HMF levels and AA. Thus, each model system was prepared once.

5-HMF and GA were analyzed at 0, 20, 40, 60, 120, 180, 240, 300 and 360 min of reaction time. 5-HMF and GA were determined immediately after sample withdrawn. Browning and AA development were measured at 0, 60, 120, 180, 240 and 360 min of reaction time, in aliquots stored for up to 2 weeks at −18 °C.

### 3.3. 5-Hydroxymethylfurfural and Gallic Acid Analysis

5-HMF and GA were analyzed by HPLC, through adaptations of the method proposed and validated by Albouchi & Murkovic [12], using a Shimadzu system (Kyoto, Japan) equipped with a quaternary pump (LC-10Advp), a column oven (CTO-10Asvp), a manual sample injector (8125 Rheodyne valve equipped with a 20 µL loop), a degasser (DGU-14A) and a diode array detector (SPD M10Avp). Chromatographic separation was achieved in 10 min using a C18 reversed-phase column (SUPELCO, 250 mm × 4.6 mm, 5 µm) kept at 40 °C and isocratic elution with 0.3% aqueous formic acid and methanol (90:10), at flow rate of 1.0 mL/min. 5-HMF and GA were identified by comparison of their retention times and UV spectra with those of commercial standards. Quantification was performed by external standardization using 280 nm and 220 nm for 5-HMF and GA, respectively. Data were acquired by LCMS solutions software (version 2.04, 2003, Shimadzu Corporation, Kyoto, Japan). Analyses were carried out in duplicate and 5-HMF and GA results were expressed as µg/mL.

### 3.4. Color Development (Browning)

Browning of model systems was measured at 420 nm using an UV-spectrophotometer (UV-1800, Shimadzu, Japan) after proper dilution of samples and using ultrapure water as blank [37].

### 3.5. Antioxidant Activity Analysis

AA of model systems was determined by FRAP (Ferric Reducing Antioxidant Power) and TEAC (Trolox Equivalent Antioxidant Capacity) assays, as described by Moura-Nunes et al. [38]. FRAP assay was slightly modified using 4 min of reaction time and Trolox as standard. All analyses were performed in triplicate and results were expressed as mmols of Trolox/L.

### 3.6. Statistical Analysis

5-HMF, color and AA developments were evaluated by calculating the area under curve (AUC) using initial values as baseline and considering “peaks” that go below the baseline. To estimate the standard deviation of AUC values of 5-HMF, browning and AA, a 10% coefficient of variation was used, chosen as twice the value found in the reproducibility test described in item 2.2. Student’s unpaired *t*-test was used to evaluate the effect of gallic acid addition on 5-HMF, color and AA developments. These statistical analyses were performed using GraphPad Prism software for Windows (version 6.01, GraphPad Software, San Diego, CA, USA). Pearson’s correlation analysis was performed by Statistica software (version 7.0, StatSoft Inc., Tulsa, OK, USA) with a confidence interval of 95%.

## 4. Conclusions

In thermally processed foods, it is very difficult to mitigate 5-HMF formation without compromising browning and thus food sensory acceptability. The preventive strategy of adding GA to inhibit 5-HMF formation can be studied using simplified Maillard model systems, which allow control of reaction conditions. GA participated in the MR rather than being thermally degraded, causing the same effect (either negative or positive, depending on the sugars and amino acids involved) on the formation of both colored and antioxidant compounds. Although GA caused diverse effects on 5-HMF development, the intent of mitigating its formation without compromising browning and AA development was achieved by adding GA in glucose/arginine and sucrose/arginine model systems, possibly through its dicarbonyls-trapping capacity. In these systems, approximately half of the 5-HMF formation was mitigated, while color development has doubled.

## Data Availability

The datasets generated for this study are available on request to the corresponding author.

## Supplementary Materials

The following supporting information can be downloaded online: Figure S1: Neither GA concentration (●) nor its antioxidant activity measured by FRAP assay (▲) changed in the blank system after 360 min (*p* > 0.05, ANOVA followed by Tukey’s post hoc test). The blank system was prepared by heating a gallic acid solution under the same conditions used for model systems.

## Sample Availability

Samples of the compounds are not available from the authors.
