# Supplementary material for: Gallic Acid Mitigates 5-Hydroxymethylfurfural Formation while Enhancing or Preserving Browning and Antioxidant Activity Development in Glucose/Arginine and Sucrose/Arginine Maillard Model Systems
Source: Molecules. 2022 Jan 27;27(3):848. doi: 10.3390/molecules27030848 (PMC8838868; doi:10.3390/molecules27030848)
Supplement: Supplementary file 1 [file molecules-27-00848-s001.zip › molecules-1557063-supplementary.pdf]

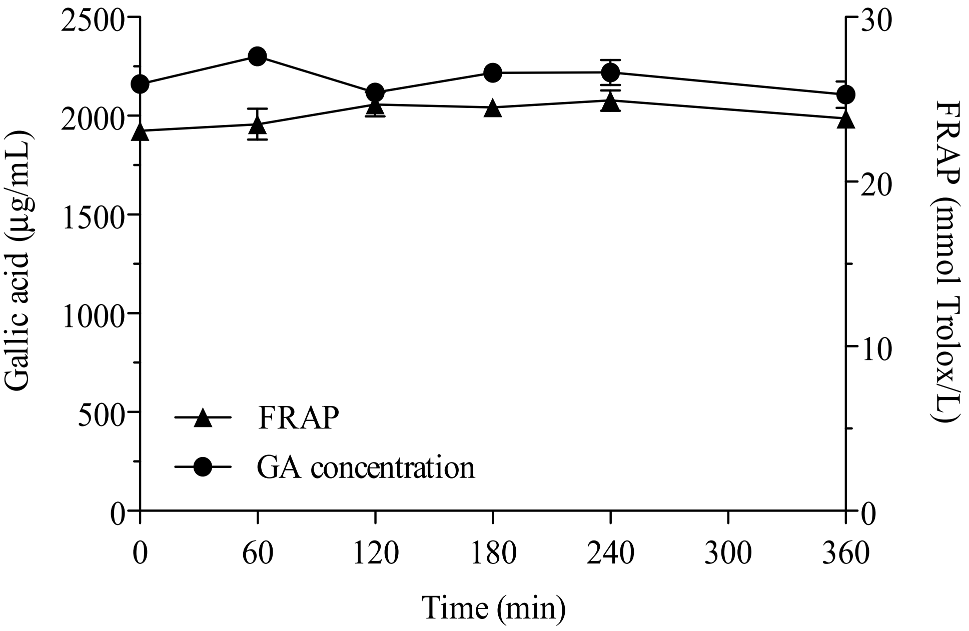

Supplementary Figure S1. Neither GA concentration (●) nor its antioxidant activity measured by FRAP assay (▲) changed in the blank system after 360 min ($p > 0.05$, ANOVA followed by Tukey's *post hoc* test). The blank system was prepared by heating a gallic acid solution under the same conditions used for model systems.
